# Clinically relevant pseudoexons of the *GALNS* gene and their antisense-based correction

**DOI:** 10.1186/s10020-025-01243-0

**Published:** 2025-05-17

**Authors:** Igor Bychkov, Elza Shchukina, Ekaterina Zakharova

**Affiliations:** Department of Molecular Mechanisms of Inherited Metabolic Disorders, Laboratory of Experimental Gene Therapy for Inherited Metabolic Disorders, Research Centre for Medical Genetics, Moscow, Russia

**Keywords:** Antisense oligonucleotides, Antisense therapy, Gene therapy, Cryptic exons, Poison exons, Aberrant splicing, Modified U7 small nuclear RNA, Circular RNA, mU7snRNA, Morquio A syndrome

## Abstract

**Background:**

Biallelic pathogenic variants in the *GALNS* gene lead to Mucopolysaccharidosis Type IVA (MPS IVA), a rare lysosomal storage disorder. *GALNS* encodes the enzyme N-acetylgalactosamine-6-sulfatase, whose deficiency causes accumulation of glycosaminoglycans and leads to a broad spectrum of clinical manifestations primarily affecting the osteoarticular system. Several studies have shown that, in 10%–15% of patients with the biochemical phenotype of MPS IVA, standard molecular genetic testing fails to identify one or both causative variants in the *GALNS* gene.

**Methods:**

We performed an in-depth investigation of *GALNS’* splicing, with a special focus on deep-intronic mutations that lead to activation of pseudoexons (PEs). Using bioinformatic tools, we analyzed all deep-intronic variants in *GALNS* available in public databases and subjected the most relevant ones to in vitro analyses using minigenes.

**Results:**

We characterized eight PE-activating variants, one of which (c.121-210C > T) represents a recurrent pathogenic variant which has long been hidden behind the mask of a polymorphic variant. In addition, we demonstrate that *GALNS’* splicing can produce a diverse range of mRNA isoforms containing so-called wild-type PEs, which are present at low levels as part of non-productive splicing, and weak canonical exons which are prone to skipping. We show that PE-activating variants cluster within wild-type PEs, highlighting the need for closer scrutiny of these regions during genetic testing.

Finally, we applied modified U7 small nuclear RNAs and circular RNAs to efficiently block the identified PEs and pave the way for personalized antisense-based therapy for MPS IVA patients.

**Conclusion:**

The results of this study expand the understanding of *GALNS* gene splicing, indicating hotspots for splicing mutations. The presented data not only help to increase the diagnostic yield for MPS IVA but also unveil new therapeutic approaches for a number of MPS IVA patients.

**Supplementary Information:**

The online version contains supplementary material available at 10.1186/s10020-025-01243-0.

## Background

Mucopolysaccharidosis Type IVA (MPS IVA, OMIM 253000) is an inherited lysosomal storage disorder caused by a deficiency of the N- acetylgalactosamine-6-sulfate sulfatase enzyme (GALNS). GALNS is involved in degradation of glycosaminoglycans—keratan sulfate and chondroitin-6-sulfate, whose excessive accumulation in tissues leads to a wide range of clinical presentations (Sawamoto, et al. 2020). The osteoarticular system is the predominantly affected, with the first symptoms appearing within a few years of age. They include dwarfism due to skeletal dysplasia, spinal cord compression, tracheal obstruction, joint laxity, kyphoscoliosis, pectus carinatum, coxa valga, and genu valgum (Montano et al. 2007; Hendriksz et al. 2013; Hendriksz et al. 2015). Connective tissue damage leads to pulmonary insufficiency, heart disease, hearing loss, and ocular changes.

Currently available therapies for MPS IVA include enzyme replacement therapy and hematopoietic stem cell transplantation (Sawamoto, et al. 2020; Akyol et al. 2019). In most cases of MPS IVA, the skeletal dysplasia progresses rapidly and leads to irreversible changes, whereas mild forms can remain unnoticed for many years (Yi et al. 2024). As the current therapies cannot reverse the damage to the osteoarticular system, early and definitive diagnosis of MPS IVA is crucial for managing the disease progression. The diagnosis of MPS IVA is established after detection of the reduced GALNS activity in leukocytes or fibroblasts, followed by the identification of biallelic variants in the *GALNS* gene (Sawamoto, et al. 2020).

To date, about 600 unique variants in the *GALNS* gene have been registered in the Human Gene Mutation Database (http://www.hgmd.org), most of which are missense (Zanetti et al. 2021). The molecular genetic diagnostics of MPS IVA is usually performed by Sanger sequencing of the *GALNS* gene exons or by NGS panels sequencing. Several studies have reported that in 10%—15% of patients one or both causative variants are missed during routine molecular genetic testing (Zanetti et al. 2021; Yi et al. 2022; Morrone et al. 2014). To improve the diagnostic yield, researchers implemented RNA analysis and identified reduced *GALNS* expression or alterations in mRNA isoforms (Sohn et al. 2022; Caciotti et al. 2018). With the development and widespread implementation of whole genome sequencing in diagnostics, the study of mutations located deep within the introns of genes has become increasingly important (Vaz-Drago et al. 2017; Petersen et al. 2022). The majority of these mutations lead to alteration of gene splicing by activating pseudoexons, which in turn can cause the shift of mRNA reading frame, form a premature stop codon and induce nonsense mediated mRNA decay mechanism. Computational prediction of human deep-intronic variation greatly supports the identification of such variants, although confirmation of their deleterious effects on gene expression requires additional functional studies (Barbosa, et al. 2022). In addition, the accumulating data on background or non-productive splicing also supports the identification of hotspots for such variants, which warrant special attention during the analysis of sequencing data (Petersen et al. 2022; Felker et al. 2023; D, A.,, et al. 2022).

The majority of deep-intronic pathogenic variants lead to the activation of pseudoexons (PEs), which are highly relevant and easy targets for personalized antisense-based therapy. Their location within the introns of genes facilitates the design of allele-specific antisense molecules (AMs) which do not interfere with normal splicing. Wild-type PEs, which are present at low levels as a part of non-productive splicing, and poison exons, which are normally involved in the regulation of gene expression, can also be targeted by AMs to upregulate gene expression or even to restore splicing altered by the variants located in the neighboring exons (Spangsberg Petersen et al. 2024; Lim et al. 2020; Kuijper et al. 2021).

The field of antisense therapy has developed a variety of effective strategies to modulate splicing. Beyond traditional methods relying on the delivery of chemically modified antisense oligonucleotides, researchers are increasingly employing modified U7 small nuclear RNAs (modU7snRNAs) (Lesman et al. 2021; Gadgil and Raczynska 2021) and circular RNAs (circRNAs) (Ren et al. 2023) as carriers for antisense molecules. These approaches enable efficient delivery of AMs to a wide range of tissues via adeno-associated viral vectors, thereby providing additional therapeutic possibilities for MPS IVA patients.

## Materials and methods

### Variant selection

Variants were named according to the *GALNS* reference sequence NM_000512.5 and GRCh38.p14 (hg38) genome assembly.

The c.423-862C > T and the c.1003-1570G > T variants were identified in our patients with biochemical phenotype of MPS IVA during molecular genetic testing (Supplementary information 1). The remaining variants were selected after analysis of three main public repositories of the *GALNS* gene variants: ClinVar (https://www.ncbi.nlm.nih.gov/clinvar/, assessed at Aug 2023), The Human Gene Mutation Database (https://www.hgmd.cf.ac.uk/ac/, assessed at Aug 2023) and gnomAD v2.1.1 (https://gnomad.broadinstitute.org/, assessed at Aug 2023).

The selection criteria were:

1. The variant is a single nucleotide variant located at a distance of at least 100 bp (optimal length of the minimal intron) from annotated for NM_000512.5 exons.
2. The variant is predicted to create or strengthen the splice site. The SpliceAI (Jaganathan, et al. 2019) delta score (DS) for acceptor gain or donor gain for the variant is greater than 0.5 (the threshold, recommended by the original article). We also included 7 variants with 0.2 < DS < 0.5 as they are located within the same minigenes. For clarity, splice site strength was also calculated using MaxEntScan (Yeo and Burge 2004).
3. The variants with high population frequencies (> 1%) were excluded, except the c.121-210C > T variant, which has the minor allele frequency of 3% in African/African American populations. This variant was suspected to be a complex allele and his effect on splicing was tested in combination with the neighbor c.208A > G variant.

The final list of variants with detailed information is presented in Table S1.

### Construction of minigenes

Minigenes were created using the scaffold of the pSPL3_Flu2_mTK vector, as described previously (Bychkov et al. 2022). Minigenes were designed to include the studied variants with at least 200 bp flanking the predicted boundaries of PEs. For all constructs except Minigene 1, the neighboring exon (or multiple exons, if located within 5000 bp) was included to better reconstitute the wild-type splicing. Fragments of the *GALNS* gene were cloned into the multiple cloning site within the intron of the pSPL3_Flu2_mTK vector located between two constitutively spliced exons, V1 and V2.

The studied variants were introduced using Phusion Site-Directed Mutagenesis Kit (Thermo Fisher Scientific, Waltham, MA, USA) and the resulting plasmids were sequenced to confirm the absence of amplification errors. In total, five constructs were created.

HEK293T cells (ATCC number: CRL-3216) were transfected with minigene plasmids at 80% confluency in 24-well plates using 1,5 μL of TurboFect Transfection Reagent (Thermo Fisher Scientific, Waltham, MA, USA). After 48 h, RNA was extracted and reverse transcribed. The plasmid-specific cDNA was amplified with primers located within exons V1 and V2.

### RNA analysis

RNA was extracted using ExtracRNA reagent (Evrogen, Moscow, Russia). cDNA was synthesized using 5X RT MasMIX (Dialat, Moscow, Russia) and oligo(dT) primers. Sanger sequencing and fragment analysis were performed on ABI PRISM 3500xL Genetic Analyzer (Thermo Fisher Scientific, Waltham, MA, USA). Minigene-specific primers located in exons V1 and V2, with 5’-end 6-FAM modification, were used to amplify the splicing products by PCR using SmarTaq polymerase (Dialat, Moscow, Russia).

The amplicons were further visualized by 3% agarose gel electrophoresis and subjected to fragment analysis. The results of fragment analysis were quantified and visualized using Coffalyser.Net software (https://www.mrcholland.com/technology/software/coffalyser-net). In cases where multiple RNA isoforms were present in the sample, the PCR product was ligated into pAL2-T vector (Evrogen, Moscow, Russia). E. coli cells were transformed with the ligation product and seeded on agar plates for subsequent PCR of individual bacterial colonies. Triplicates of PCR products with different lengths were then selected for Sanger sequencing to validate all RNA isoforms detected by fragment analysis.

### Design of antisense molecules

To block the inclusion of PEs, we used antisense molecules (AMs) expressed as modified U7 small nuclear RNA (modU7snRNA) and circular RNA (circRNA). ModU7snRNA and circRNAs cassettes were designed as described previously (Bychkov et al. 2025) and cloned into the pcDNA3.1 vector (https://www.addgene.org/vector-database/2092/) between XhoI and BglII restriction sites.

The main criteria for designing modU7snRNA antisense sequences were:

1. For blocking of PEs, modU7snRNAs were designed to target splice sites and motifs of exonic splicing enhancers. To identify these motifs, exons with 50 bp of adjacent introns were analyzed in HExoSplice web interface (http://bioinfo.univ-rouen.fr/HExoSplice_submit/connexion.php, assessed at Aug 2023) and ESEfinder3.0 (https://esefinder.ahc.umn.edu/cgi-bin/tools/ESE3/esefinder.cgi, assessed at Aug 2023). The SC35 motifs with high scores were considered as the preferred targets for AMs.
2. The accessibility of modU7snRNA target sites was determined by modeling the RNA secondary structure with RNAfold v2.4.18 (http://rna.tbi.univie.ac.at/cgi-bin/RNAWebSuite/RNAfold.cgi) and mFold (http://www.unafold.org/mfold/applications/rna-folding-form.php). Exons with 50 bp of adjacent introns were analyzed with standard settings to identify open and partially open regions of RNA. AMs were designed to preferably overlap with these regions.
3. The first screening step of AMs included antisense sequences with length between 25 and 30 bp to identify as many AM-sensitive regions as possible. In the next step, the lengths of the most efficient AMs were reduced to 18–20 bp to improve the specificity.

## Results

### Overview of the studied variants

**Fig. 1:**
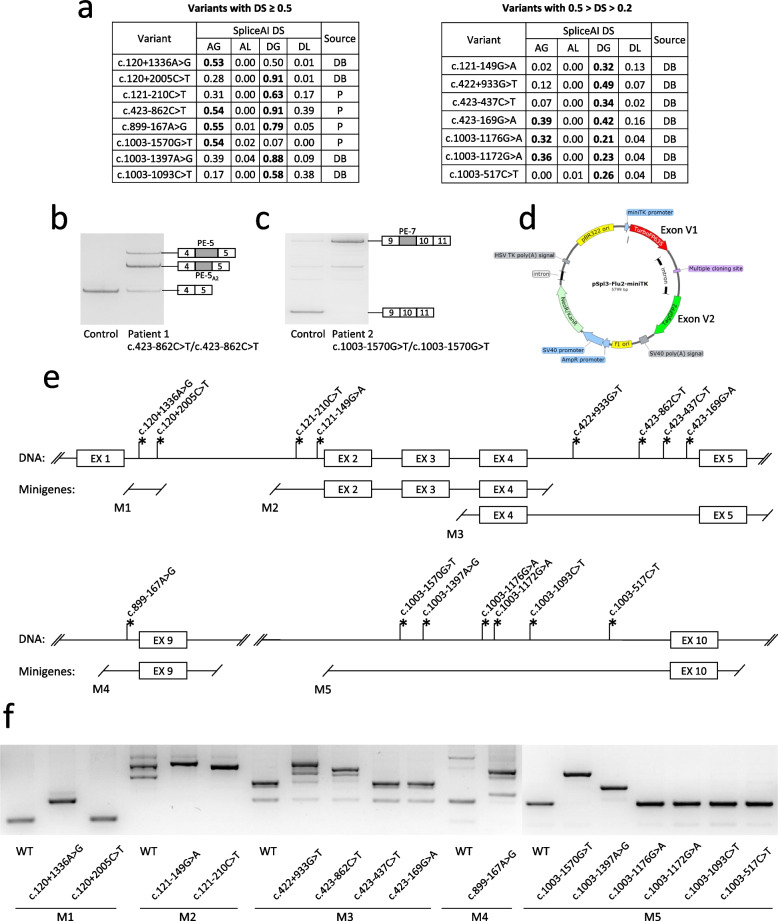
Overview of the studied variants and minigene assay. **a**—Classification of the studied variants based on predictive scores. The main group consists of variants with high predictive scores (DS ≥ 0.5), while the additional group comprises variants with medium predictive scores (0.2 < DS < 0.5) located in the vicinity. SpliceAI: DS – delta score, AG/AL – acceptor splice site gain/loss, DG/DL – donor splice site gain/loss. Source: P – the variant was identified earlier in patients with MPS IVA, DB – the variant was registered in mutation databases without association with MPS IVA patients. **b**, **c** –RNA analysis results of patients’ white blood cells samples. In both cases, additional high molecular weight isoforms were detected, which turned out to be PEs, activated by the c.423-862C > T and the c.1003-1570G > T variants, respectively. **d** –structure of the minigene vector pSpl3-Flu2-miniTK. e – scheme of the studied genomic loci cloned into the multiple cloning site of pSpl3-Flu2-miniTK. f – the results of HEK293T cells transfection with wild-type and mutant minigenes. Minigene-specific splicing products were amplified and visualized by 3% agarose gel electrophoresis

According to the criteria specified in the Materials and methods section, we selected 15 deep-intronic *GALNS* variants for in vitro splicing analysis using minigenes. In brief, the variants were selected after bioinformatic analysis of public repositories of *GALNS* gene variants and divided into two groups based on high (DS ≥ 0.5) and medium (0.5 > DS > 0.2) SpliceAI predictive scores (Fig. 1a). Two variants, c.423-862C > T and c.1003-1570G > T were also identified in our patients with a biochemical phenotype of MPS IVA after RNA analysis (Fig. 1b, c and Supplementary information 1).

Minigenes were created by placing the studied genomic loci into the intron of the pSpl3-Flu-miniTK vector between two constitutively spliced exons V1 and V2 (Fig. 1d, e). HEK293T cells were transfected with the minigene plasmids, and RNA was extracted after 48 h for analysis of minigene-specific splicing products. Visualization and fragment analysis of PCR products demonstrated a clear difference between wild-type and mutant minigenes for eight variants: c.120 + 1336A > G, c.121-210C > T, c.121-149G > A, c.422 + 933G > T, c.423-862C > T, c.899-167A > G, c.1003-1570G > T, and c.1003-1397A > G (Fig. 1f). These variants were further subjected to the detailed analysis and the testing of antisense splicing-modulating molecules against corresponding PEs. SpliceAI, still being the state-of-the-art tool for splice-altering variants identification, correctly predicted the PE activation for 6 of 8 high-scored variants (DS ≥ 0.5) and for 2 variants with medium score (0.5 > DS > 0.2).

### Terminology and annotation of pseudoexons

A strict definition of the term “pseudoexon” has not yet been fully established. “Pseudoexon”, “cryptic exon” and “poison exon” are three main terms used in the literature to describe roughly the same phenomenon. The term “poison exon” is used to describe an alternative exon containing a premature stop codon, by emphasizing its well-characterized regulatory function in gene expression (in case of inclusion in mRNA, it downregulates gene expression by activation of nonsense-mediated mRNA decay mechanism). In contrast, “pseudoexon” and “cryptic exon” are used interchangeably to describe a fragment of a gene that lacks any known functional role but can be present in small amounts in mature RNA as a part of non-productive splicing or in a significant amount as a consequence of mutations or various cell conditions which dysregulate splicing. Additionally, if the presence of a PE in the mRNA is not associated with the studied splice-altering variants, we call it “wild-type PE”.

Some of the identified PEs have more than one isoform, resulting from the utilization of different acceptor or donor splice sites. To simplify the annotation of PEs, we define them according to the longest isoform and name shorter isoforms according to their alternative splice sites. For example, PE-1 is the longest isoform, PE-1_D2_ is the shorter isoform resulting from an alternative donor splice site, and PE-1_A2_ results from an alternative acceptor splice site.

The main transcript of the *GALNS* gene (Ensemble transcript: ENST00000268695.10, NCBI Reference Sequence: NM_000512.5) consists of 14 exons, some of which, as described further, are “prone to skipping”. We avoid the term “alternative exon”, as it is usually used in the context of productive alternative splicing, which gives rise to significant amounts of different functional mRNA isoforms.

### Background splicing of the *GALNS* gene

Background splicing (BS) refers to minor splicing events caused by cryptic splice site usage, exon skipping or PE activation. Myriads of these events represent a consequence of insignificant imperfect splicing regulation under normal conditions, or dysregulation of splicing during various pathological conditions, including stress, cancer, and aging, while some part represents tissue specific splicing patterns. The information on *GALNS’* BS was obtained from bulk RNA-seq data from about 44 000 RNA samples of various cell types and of various experimental conditions deposited in the National Center for Biotechnology Information Sequence Read Archive (SRA) (Leinonen, et al. 2011).

**Fig. 2:**
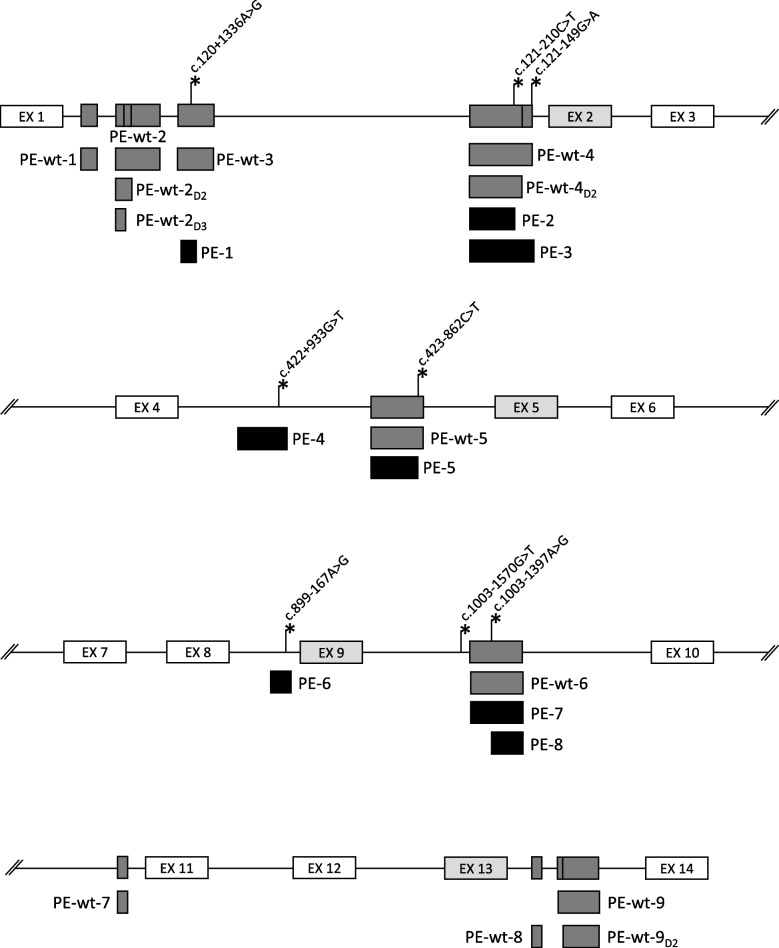
Overview of background splicing of the *GALNS* gene. The schematic representation of *GALNS* gene fragments at the DNA level, indicating locations of the studied splice-altering variants and activated PEs (black), wild-type PEs (dark grey), and “weak” canonical exons (light gray)

**Fig. 3:**
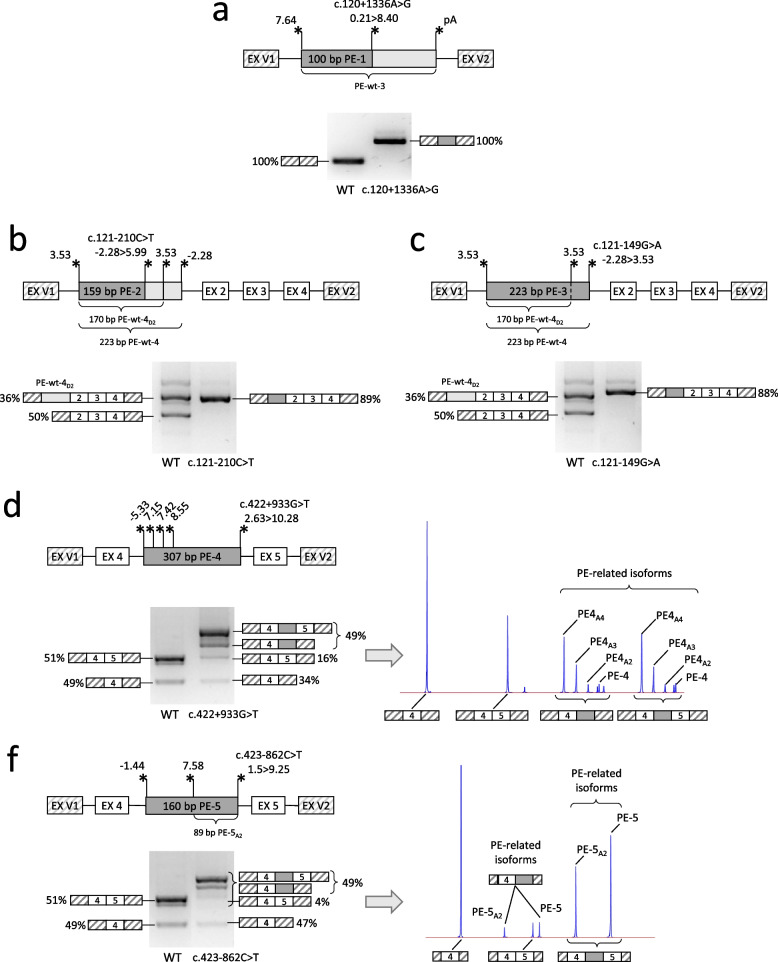
Detailed analysis of the studied variants. **a**-**f—**schemes of the genomic loci incorporated into minigene vectors and visualization of minigene-specific splicing products are shown for each variant. The mean amounts of transcript isoforms from three biological replicates are quantified by fragment analysis and are shown as percentages of all isoforms (an isoform is shown if it constitutes more than 5%). For the c.422 + 933G > T and the c.423–862 variants, the electropherograms are also shown to better represent all of the isoforms (the height of the fluorescence peak reflects the quantity of the isoform). EX V1 and EX V2 – two constitutively spliced exons of the minigene vector. Asterisks indicate splice sites with their relative strength (MaxEntScan score) and polyadenylation signal—pA

**Fig. 4:**
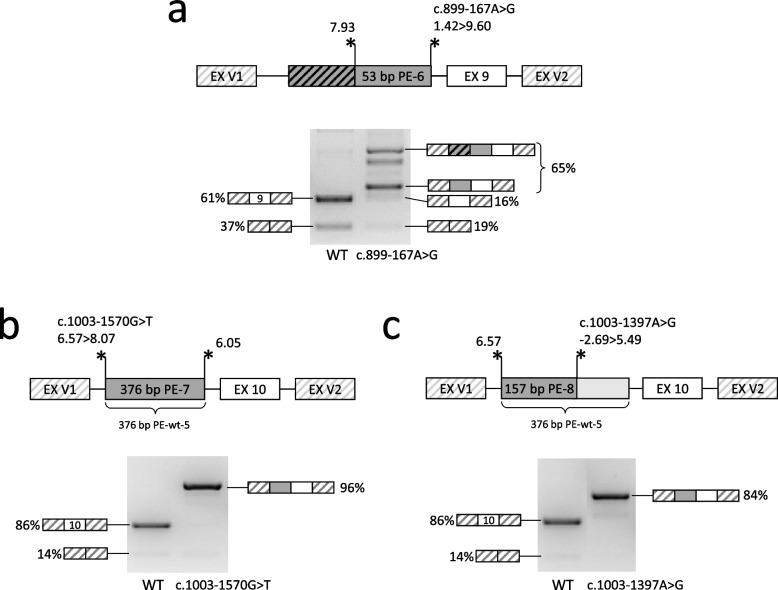
Detailed analysis of the studied variants. **a**-**c -**schemes of the genomic loci incorporated into minigene vectors and visualization of minigene-specific splicing products are shown for each variant. The mean amounts of transcript isoforms from three biological replicates are quantified by fragment analysis and are shown as percentages of all isoforms (an isoform is shown if it constitutes more than 5%). The crossed gray bar – retention of the intron fragment upstream of PE-6 (the minigene artifact). EX V1 and EX V2 – two constitutively spliced exons of the minigene vector. Asterisks indicate the splice sites with their relative strength (MaxEntScan score)

Using Snaptron (https://snaptron.cs.jhu.edu/) (Wilks et al. 2018) and SRAv2 data we identified the main BS events in the *GALNS* gene (20 of 118 events with the highest amounts of discordant reads). These events represent nine wild-type PEs and skipping of exons 2, 5, 9 and 13 (Fig. 2). Detailed information about BS, including RNA-seq reads distribution and coordinates of the PEs, is presented in Fig. S1. As expected, 7 of the 8 identified splice-altering variants are located within the bodies or splice sites of wild-type PEs or near exons prone to skipping (Fig. 2). Skipping of weak exons 2, 5, and 9, as well as inclusion of wild-type PE-wt-4_D2,_ was also observed in our minigene assay (Figs. 3 and 4), while the inclusion of PE-wt-6 was observed in the control sample of white blood cells RNA (Fig. 1c).

### The detailed analysis of the studied variants

#### The c.120 + 1336A > G variant

The c.120 + 1336A > G variant is located in the dense cluster of PEs near the exon 1 and within the body of wild-type PE-wt-3, the terminal exon of ENST00000568311.1 transcript (Fig. 2). The variant has high predictive score (DS 0.53) and is also presented in Splice-Site Creating Variants Data Base (SSCV DB v1.0, https://sscvdb.io/) as the variant leading to “cryptic exon inclusion”. However, the variant is presented in heterozygous state in 36 individuals and in homozygous state in 1 individual from gnomAD v4.1.0 database, which casts doubt on its pathogenicity.

The results of the minigene assay demonstrated that c.120 + 1336A > G is clearly a spliceogenic variant. The variant creates strong donor splice site and, utilizing the strong acceptor splice site of PE-wt-3, leads to the formation of 100 bp PE-1, with a complete absence of the correct transcript (Fig. 3a). The inclusion of PE-1 introduces a premature stop codon.

#### The c.121-210C > T and the c.121-149G > A variants

The c.121-210C > T and the c.121-149G > A variants are located within the body of PE-wt-4 near the exon 2. A reliable acceptor splice site and two closely located donor splice sites of this PE give rise to the 223 bp PE-wt-4 and the 170 bp PE-wt-4_D2_, both of which contain a premature stop codon (Figs. 2 and 3b). Notably, an additional 53 bp of PE-wt-4 represents a duplication of the 3’ end of PE-wt-4_D2_ with only 5 mismatches, one of which (c.121-149G) weakens the donor splice site of PE-wt-4 (Fig. 3b). This issue complicates the alignment of RNA-seq reads in this area, however according to Snaptron analysis of SRAv2 data, these PEs are expressed on average at approximately the same level (Fig. S1). Despite this, PE-wt-4_D2_ is predominantly observed in our minigene assay and is annotated as a part of ENST00000568613.5 transcript.

The c.121-210C > T variant has a high predictive score (DS 0.63) and is registered in the ClinVar database as a variant with “conflicting classifications of pathogenicity” (Variation ID: 1048373). Several studies have identified this variant in patients with MPS IVA but failed to interpret it as disease-causing due to its high allele frequency of 3% in the African/African American population (Zanetti et al. 2021; Sohn et al. 2022; Chuang, et al. 2022).

The results of minigene assay demonstrated that the c.121-210C > T variant creates a strong donor splice site and leads to the inclusion of the 159 bp PE-2 in the vast majority of mRNA molecules (Fig. 3b). In our previous work, we demonstrated that the reason for its high frequency is the protective c.121-208A > G variant located in the vicinity (Igor Bychkov et al. 2023). When presented in cis, c.121-208A > G inactivates strong splice site created by c.121-210C > T and maintains the correct splicing. We demonstrated that both variants are always presented in cis in analyzed individuals from gnomAD database, while five probands with MPS IVA from unrelated families lack the protective c.121-208A > G variant (Igor Bychkov et al. 2023). In addition, PE-2 was previously identified during mRNA analysis of one of the above-mentioned probands and of one proband from another study (Yi et al. 2022; Sohn et al. 2022). Together, this data strongly supports the pathogenicity of the c.121-210C > T variant.

The c.121-149G > A variant, located in the vicinity, was found in two heterozygous individuals from the gnomAD database and has a medium predictive score (DS 0.32), although it significantly increases the strength of donor splice site of PE-wt-4, making it identical to PE-wt-4_D2_ (Fig. 3c). The results of minigene assay demonstrated that c.121-149G > A leads to the complete absence of the correct transcript and inclusion of the 223 bp PE-3 in the vast majority of mRNA molecules.

#### The c.422 + 933G > T and the c.423-862C > T variants

Among the variants located in the intron 4, only c.422+933G > T and c.423-862C > T demonstrated significant alteration of splicing in the minigene assay (Fig. 1f). The wild-type minigene encompassing the studied locus demonstrated partial skipping of exon 5 (defined as “weak”) and 51% of the correct transcript (Figs. 3d, f).

The c.422 + 933G > T variant was found in 1 heterozygous individual from the gnomAD database and has a medium predictive score (DS 0.49). The variant creates a strong donor splice site and leads to the formation of several PEs, named PE-4 (307 bp), PE-4_A2_ (290 bp), PE-4_A3_ (268 bp), and PE-4_A4_ (246 bp), which differ in their acceptor splice sites (Fig. 3d). All PEs introduce a premature stop codon and lead to reduction of the correct transcript from 51 to 16% (3.2-fold).

The c.423-862C > T variant was previously associated with altered *GALNS* splicing in a patient with a severe form of MPS IVA and a single identified variant, c.697G > A (p.Asp233Asn) (Caciotti et al. 2018). During molecular genetic testing of patients with biochemical phenotype of MPS IVA, we identified two additional cases where the c.423-862C > T variant was found in a homozygous state and in a heterozygous state with c.131G > T (p.Gly44Val). Fresh blood was obtained from the homozygous patient and analysis of RNA from white blood cells was performed. Amplification of the *GALNS* cDNA revealed two additional high molecular weight isoforms, which turned out to be overlapping PEs (Fig. 1b).

The c.423-862C > T variant has a high predictive score (DS 0.91) and is absent from the gnomAD database. The variant is located within the body of PE-wt-5, which is the first exon of the corresponding transcript as it lacks acceptor splice site and has only weak donor splice site downstream of c.423-862C > T. The c.423-862C > T variant creates strong donor splice site and leads to formation of two PEs, named PE-5 (160 bp) and PE-5_A2_ (89 bp), which utilize different acceptor splice sites (Fig. 1b). This splicing pattern was also observed in minigene assay (Fig. 3f). The fragment analysis of minigene-specific splicing products demonstrated that both PEs are expressed at approximately the same level and lead to reduction of the correct transcript from 51 to 4% (12.8-fold). Notably, the acceptor splice sites of PE-4 and PE-5 are both suboptimal (MaxEntScore -5.33 and -1.44 respectively) but still functional. However, unlike the PE-4, which is expressed in barely detectable amounts relative to all PE-4-related isoforms (Fig. 3d), the PE-5 is the major isoform expressed in the minigene assay (Fig. 3f) and is also presented in significant amounts RNA from white blood cells (Fig. 1b).

#### The c.899-167A > G variant

The c.899-167A > G variant was identified earlier in a heterozygous state with the c.463G > A (p.Gly155Arg) variant in a patient with a severe form of MPS IVA (Caciotti et al. 2018). The authors performed RNA analysis and identified a 53 bp PE (referred herein as PE-6) in intron 8, which is activated by the c.899-167A > G variant. The c.899-167A > G variant creates a strong donor splice site, has a high predictive score (DS 0.79) and is absent from the gnomAD database. Inclusion of PE-6 results in a frameshift and formation of a premature stop codon.

The c.899-167A > G variant is located near the exon 9, which was defined as “weak” and is present in 61% of mRNA isoforms in our minigene assay (Fig. 4a). Activation of PE-6 led to reduction of the correct transcript from 61 to 16% (3.8-fold). An additional 310 bp PE was detected in 23% of mRNA isoforms, which turned out to be a minigene artifact, as it represents the retention of an intron fragment between PE-6 and a cryptic acceptor splice site located within the intron of the minigene vector (Fig. 4a).

#### The c.1003-1570G > T and the c.1003-1397A > G variants

The c.1003-1570G > T variant was identified in a homozygous state in our patient with a severe form of MPS IVA. The variant has a high predictive score (DS 0.54) and is absent from the gnomAD database. The c.1003-1570G > T variant is located in the polypyrimidine tract of PE-wt-6, which is observed in small amounts in control sample of RNA from white blood cells (Fig. 1c). The variant slightly increases the strength of the acceptor splice site of PE-wt-6 (the isoform from the mutant allele is hereinafter referred to as PE-7), which, in turn greatly increases its inclusion and leads to the almost complete absence of the correct transcript in the both patient’s sample and the minigene assay (Figs. 1c and 4c). The inclusion of the 376 bp PE-7 introduces a premature stop codon.

The c.1003-1397A > G variant was found in one heterozygous individual from the gnomAD database and has a high predictive score (DS 0.88). The variant is located within the body of PE-wt-6, creates a strong donor splice site, and, by utilizing the acceptor splice site of PE-wt-6, leads to the formation of 157 bp PE-8 (Fig. 4d). This PE is present in the vast majority of mRNA molecules, leads to the complete absence of the correct transcript, and introduces premature stop codon.

### Blocking of PEs with antisense molecules

To investigate approaches to personalized antisense therapy for MPS IVA we decided to block four PEs (PE-2, PE-5, PE-6, and PE-7), which were identified in patients, with antisense molecules (AMs). For this purpose, we chose two expression systems based on modified U7 small nuclear RNAs (modU7snRNAs) and circular RNAs (circRNAs). These systems allow to deliver antisense molecules into a wide range of tissues and organs by viral vectors.

The detailed design principles are described in Materials and Methods section. In brief, modU7snRNAs were designed to have antisense sequence length of approximately 25 bp and target PEs splice sites or high-scored splicing enhancer motifs. The shorter versions of the most effective modU7snRNAs were designed to test how much their efficiency reduces with increasing specificity. Circular RNAs were recently described as an efficient tool for manipulating splicing (Ren et al. 2023). Circular RNAs could potentially serve as the more stable alternative to modU7snRNAs, as they lack free ends sensitive to cellular nucleases. For an expression of circRNAs we applied the Tornado system based on two self-cleaving ribozymes (Litke and Jaffrey 2019). Antisense sequences of various length (40–150 bp) were incorporated into circRNAs to cover the most sensitive target sites of PEs, identified after the modU7snRNAs screening. ModU7snRNAs and circRNAs cassettes were incorporated into expression vectors and co-transfected with minigenes.

As there are more than one isoform for most of the PEs, the efficiency of the tested AMs refers to the reduction of all PE-containing isoforms, which strongly correlates with restoration of the correct transcript amount.

### Blocking of the identified PEs with modU7snRNAs

**Fig. 5:**
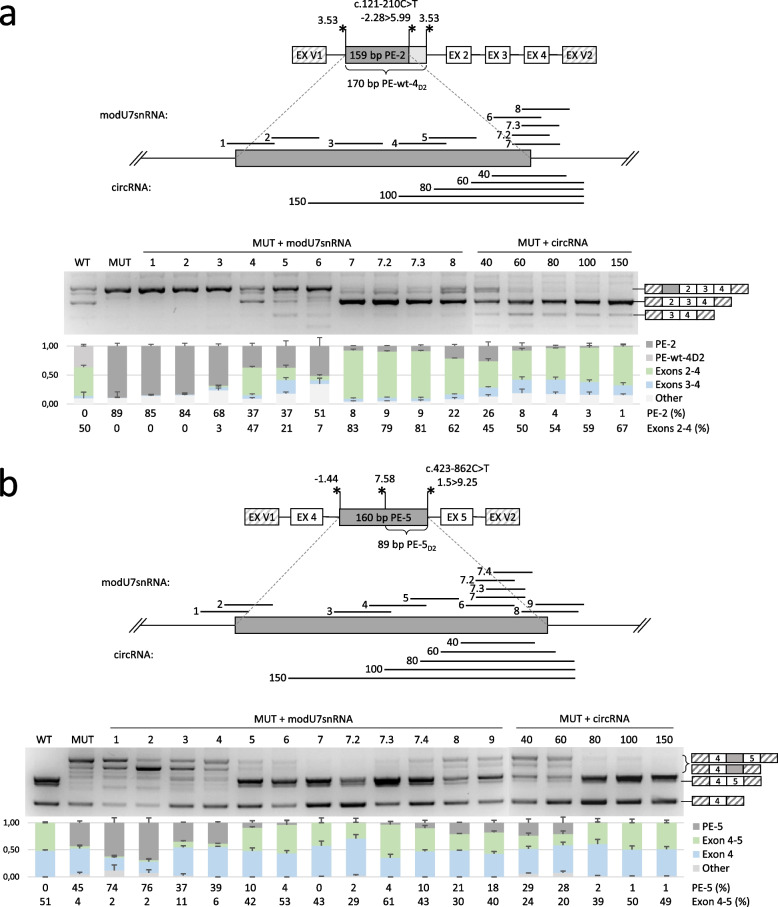
Testing of antisense molecules against PE-2 and PE-5. **a**,**b**—schemes of the genomic loci incorporated into minigene vectors and representative gel electrophoresis images of the minigene-specific splicing products are shown for each PE. PEs with adjacent 50 bp are enlarged to indicate the locations of target sites for modU7snRNAs and circRNAs. The relative amounts of transcripts are represented as stacked bar plot with means and standard deviations of three biological replicates. For clarity, maximum of three predominant transcripts are shown on the plot, and the rest are summed up and named “Other”. The PE-containing transcript is colored in dark gray, the correct transcript is colored in green, and the transcript with skipping of the neighbor exon is colored in blue

Testing of modU7snRNAs against PE-2, activated by the c.121-210C > T variant, identified the narrow region sensitive to AMs, which is located at the 3’end of the PE. The 25 bp modU7-7 efficiently blocked the PE-2 and increased the amount of the correct transcript (exons 2–4) to 83%, as it also blocks the overlapping wild-type PE-wt-4_D2_ (Fig. 5a). Its shorter 20 bp versions modU7-7.2 and modU7-7.3 demonstrated the similar efficiency.

The most effective modU7snRNAs against PE-5, activated by the c.423-862C > T variant, were also located in the narrow area near the 3’end of the PE (modU7-6, modU7-7, modU7-7.2 and modU7-7.3) (Fig. 5b). These AMs increased the amount of the correct transcript (exons 4–5) to a level comparable or even higher than in the wild-type minigene (61% of correct transcript for modU7-7.3).

**Fig. 6:**
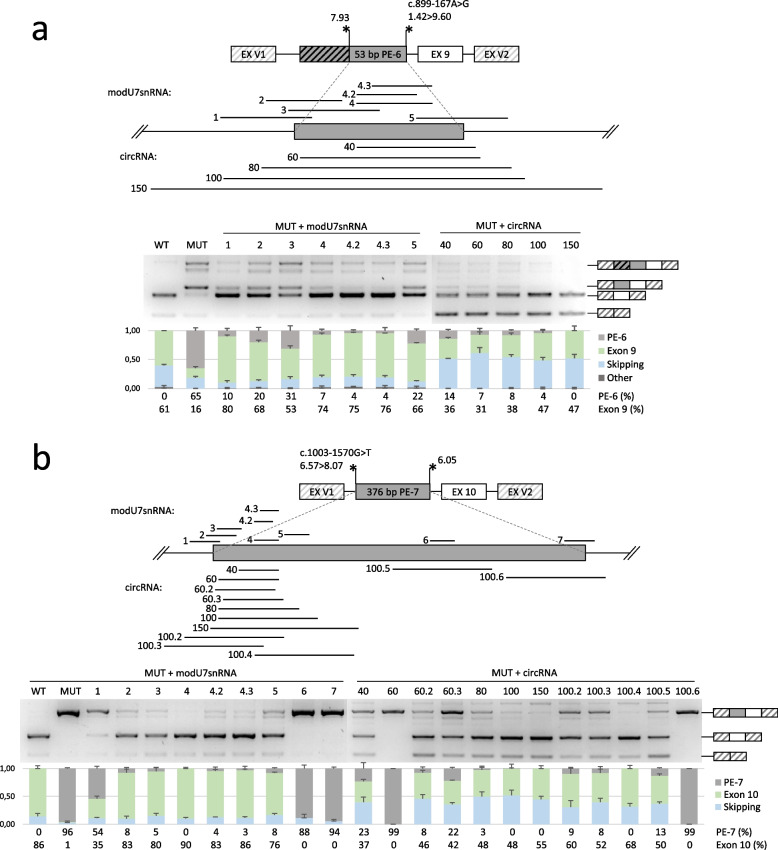
Testing of antisense molecules against PE-6 and PE-7. **a**, **b**—schemes of the genomic loci incorporated into minigene vectors and representative gel electrophoresis images of minigene-specific splicing products are shown for each PE. PEs with adjacent 50 bp are enlarged to indicate the locations of target sites for modU7snRNAs and circRNAs. The relative amounts of transcripts are represented as a stacked bar plot with means and standard deviations from three biological replicates. For clarity, a maximum of three predominant transcripts are shown on the plot, and the rest are summed up and named “Other”. The PE-containing transcript is colored in dark gray, the correct transcript is colored in green, and the transcript with skipping of the neighboring exon is colored in blue

The modU7snRNAs against PE-6, activated by the c.899-167A > G variant again demonstrated the least effectiveness when targeting PEs splice sites. The most effective AMs modU7-4, modU7-4.2, and modU7-4.3 are located within the body of the PE closer to its 3’ end and lead to a maximum of 76% of correct transcript, which is more than in the wild-type minigene (61%) (Fig. 6a).

PE-7, activated by the c.1003-1570G > T variant, represents the relatively large 376 bp PE with many relevant targets for AMs. Luckily, one of the first AMs targeting the acceptor splice site (U7-2), demonstrated high efficiency (Fig. 6b). Shifting AMs further into the PE allowed us to achieve even more efficiency with modU7-4 completely blocking the PE and leading to 90% of the correct transcript.

### Blocking of the identified PEs with circular RNAs

For clarity, circRNAs were named according to the length of their antisense sequence. In accordance with our previous results (Bychkov et al. 2025), targeting circRNAs to the PEs sites most sensitive to modU7snRNAs allowed us to identify relatively short yet efficient circRNAs. Whereas, in the original study (Ren et al. 2023), the efficient circRNAs began at an antisense sequence length of 100 bp. On average, an expected increase in the efficiency of PE blocking was observed with lengthening of antisense sequence from 40 to 80 bp, and no significant additional increase was observed from 80 to 150 bp (Figs. 5 and 6). For PE-2, PE-6, and PE-7, even a 60 bp antisense sequence almost completely blocked the corresponding PE (89% to 8%, % to %, and 96% to 8% reduction of PE-containing isoform respectively).

The notable exception was circRNA-60 for PE-7, which did not demonstrate any significant impact on splicing (Fig. 6b). Since other circRNAs targeting the same site worked as expected, we hypothesized that this specific antisense sequence might lead to strong secondary structure formation or disrupt ribozyme processing. We shifted the antisense sequence by 5 bp in both directions and created circRNA-60.2 and circRNA-60.3, which regained the ability to block the PE (reducing the PE-containing isoform from 96 to 8% and 22% respectively) and to increase the amount of the correct transcript (Fig. 6b).

To compare how efficient circRNA works when targeting sites insensitive to modU7snRNAs, we designed five additional 100 bp circRNAs against PE-7, as it is the only large PE, that allows to test long non-overlapping circRNAs. As shown in Fig. 6b, circRNA-100.6, which spans the target site insensitive for modU7snRNA (modU7-7), also failed to demonstrate any effect. This region around the donor splice site is highly enriched with SC-35 splicing enhancer motifs (Fig. S5), and the inefficiency of antisense molecules may be explained by strong local secondary structure of mRNA. CircRNA-100.5 spanning another target site insensitive for modU7snRNA (modU7.6) demonstrated high efficiency but still less than circRNA-100 spanning the most sensitive target site (modU7-4). Similarly, shifting circRNA-100 to the insensitive site for modU7-1 (circRNA-100.2, circRNA-100.3) reduced its efficiency (Fig. 6b).

It is also worth noting that for PE-2, PE-6, and PE-7, circRNAs increased the skipping of the neighboring exon (Figs. 5a, and 6a, b).

## Discussion

Characterization of intronic variants that activate PEs has become a field of rapidly growing interest (Bychkov et al. 2022; Bychkov et al. 2025; Igor Bychkov et al. 2023; Qian et al. 2021; Boisson et al. 2023; Keegan et al. 2021). Determination of their deleterious effect on gene expression and clinical significance not only increases the diagnostic yield but also unveils new targets for antisense-based therapy approaches (Tomkiewicz, et al. 2021; Reurink et al. 2023; Sangermano et al. 2019).

In this study, we utilized bioinformatic approaches to identify eight PE-activating variants in the *GALNS* gene, of which four (c.121-210C > T, c.423-862C > T, c.899-167A > G, c.1003-1570G > T) were identified in patients with MPS IVA, while the rest (c.120 + 1336A > G, c.121-149G > A, c.422 + 933G > T, and c.1003-1397A > G) are present in individuals from gnomAD database.

By the result of the minigene assay, all of the activated PEs at the mRNA level lead to frameshift and premature stop codon formation with subsequent activation of nonsense mediated mRNA decay mechanism. The residual amount of PE-containing mRNA is translated into severely truncated proteins, which lack a minimum 5 out of 14 exons of *GALNS* containing necessary functional domains. At the same time, the amount of residual wild-type mRNA can be important for the analysis of genotype–phenotype correlations and varies from 0% for PE-1, PE-2, PE-3, PE-7, PE-8, 4% for PE-5, and to 16% for PE-4, PE-6.

Among the patient’s variants, c.121-210C > T is of particular interest as it long remained hidden behind the mask of a polymorphic variant, yet revealed itself as a recurrent variant in Latin American and Chinese populations (Yi et al. 2024; Igor Bychkov et al. 2023). This variant was identified in homozygous state in patients with an attenuated phenotype of MPS IVA and could be considered a mild mutation. The c.423-862C > T variant was identified in homozygous state in a patient with an attenuated form of the disease (patient 1 from this article), which is consistent with significant residual amounts of the correct transcript in both the patient’s RNA and the minigene assay (Fig. 1b, 3f). The c.899-167A > G variant also demonstrated a significant amount of the correct transcript (16%) in the minigene assay (Fig. 4a). However, no direct genotype–phenotype correlation can be drawn, as it was identified in heterozygous state with a missense variant in a single patient with a severe form of MPS IVA. The c.1003-1570G > T variant was identified in homozygous state in a patient with the classical form of MPS IVA (patient 2 from this article), which correlates well with the complete absence of the correct transcript in patient’s RNA sample and in minigene assay (Figs. 1c and 4b). Given the presence of above-mentioned variants in patients, the sufficient criteria have been gathered to classify them as pathogenic variants (Supplementary Table 1).

The clinical significance of the remaining variants is not so obvious. The c.120 + 1336A > G variant, which is highly spliceogenic in the minigene assay and is registered in the SSCV DB as a variant leading to “cryptic exon inclusion”, is present in heterozygous state in 36 individuals and in homozygous state in 1 individual from the gnomAD v4.1.0 database. The genomic region encompassing this variant is enriched with wild-type PEs (Fig. 2), some of which contain alternative transcription start sites (PE-wt-1) or transcription termination sites (PE-wt-3). Therefore, the minigene assay may not fully reconstitute the entire spectrum of mRNA isoforms expressed in this region. For example, PE-1 activated by the c.120 + 1336A > G variant could be spliced with the alternative first exon PE-wt-1, which would maintain the reading frame of the main *GALNS* transcript starting from the exon 2. The c.422 + 933G > T variant is located within the same minigene as c.423-862C > T but leads to four times the amount of the correct transcript (16%). If we consider c.423-862C > T a mild mutation, whether such a change is sufficient for the emergence of a disease phenotype is arguable. The c.422 + 933G > T variant may represent a hypomorphic allele that can lead to a disease phenotype only if it is present in trans with a severe variant. In any case, the presented data should prompt researchers to analyze patient’s RNA when these variants are identified, while the accumulated evidence at this stage allows to classify them as likely pathogenic (Supplementary Table 1).

The most demonstrative feature of the identified PE-activating variants is their clustering within wild-type PEs or near weak canonical exons of the *GALNS* gene (Fig. 2), which is consistent with growing evidence about background splicing (BS) and its relationship with splice-altering variants. Information regarding the gene’s BS serves as a strong predictor of the outcomes of splicing variants, recursive splicing, and aberrant splicing in cancer (D, A.,, et al. 2022). Background splice sites and PEs with low inclusion rates (wild-type PEs) can also indicate hotspots for pathogenic splice-altering variants. Wild-type PEs can be activated even by a small change in the splicing code, such as the formation of a splicing enhancer motif or the disruption of a splicing silencer motif (Petersen et al. 2022). On the other hand, the splicing of weak canonical exons can be altered by the disruption of a splicing enhancer motif or the formation of a splicing silencer motif (Holm et al. 2024). Currently, this type of splice-altering variants is predicted by bioinformatic algorithms with very low efficiency and can easily escape the attention of researchers. Considering that the wild-type PEs and four weak canonical exons of the *GALNS* gene are hotspots for splice-altering variants, the appropriate bioinformatic tools, that take into account splicing regulatory elements (splicing enhancers and silencers), should be used to analyze the identified variants in these regions.

The identified PEs are certainly the relevant targets for personalized antisense therapy (Kim et al. 2019). Besides the conventional approaches that utilize chemically modified oligonucleotides, growing interest is directed at modU7snRNAs (Lesman et al. 2021; Gadgil and Raczynska 2021) and circRNAs (Ren et al. 2023) as efficient tools for splicing modification. ModU7snRNAs are widely used to stimulate exon skipping, primarily in Duchenne muscular dystrophy studies, while circRNAs are just beginning to be used for this purpose. Based on the results of a small number of studies, efficient splicing correction with circRNAs requires longer antisense sequences when directed to the same target site as modU7snRNAs (Ren et al. 2023; Bychkov et al. 2025). Thus, circRNAs are more prone to formation of secondary structures and non-specific binding, and their further use as a tool for gene therapy requires an active investigation.

Determination of the most efficient target for antisense splice-modulating molecules requires their screening at a large scale. Nevertheless, several parameters significantly affect their efficiency and increase the chance of success (Tomkiewicz, et al. 2021; Aartsma-Rus et al. 2023; Aartsma-Rus et al. 2009; Aartsma-Rus et al. 2010; Goyenvalle et al. 2023). Although these parameters have been tested primarily in exon-skipping experiments with 2’-OMe-RNAs and Morpholino oligonucleotides, most of them can be transferred to modU7snRNA-based experiments. The length of the most favorable AMs varies between studies, but ideally, a balance should be maintained between the high efficiency and low specificity of long AMs and the high specificity and relatively low efficiency of short AMs (Shimo et al. 2024). Moreover, long antisense sequences, especially those tailed with functional motifs (e.g., hnRNP A1 binding sites), are more prone to the formation of unwanted secondary structures and could inhibit the processing of modU7snRNA.

Overall, we demonstrated that a 20 bp antisense sequence length for modU7snRNA is sufficient to efficiently block all of the patients’ PEs when targeted to the most sensitive site of PE. Targeting circRNAs to the same site allows the use of relatively short antisense sequences of 60–80 bp or, in some cases, 40 bp, whereas in the original study (Ren et al. 2023), the efficient ones started with an antisense sequence length of 100 bp, that compromises their specificity. On the other hand, we demonstrated that targeting even long 100 bp circRNAs to the insensitive site of PE could completely abolish their activity, likely due to strong local secondary structures of mRNA (Fig. 6b).

In our future work, we are planning to test the identified most effective AMs in cultured fibroblasts derived from our patients. These experiments are a critical next step to demonstrate whether treatment with modU7snRNA and circular RNAs can restore *GALNS* enzyme activity and the transcriptome-wide specificity of the AMs.

Overall, we hope that the results of this study will not only increase the diagnostic yield for MPS IVA but also unveil new therapeutic approaches for a number of MPS IVA patients.

## Supplementary Information

Supplementary Material 1: Figure S1.

Supplementary Material 2: Figures S2-S5.

Supplementary Material 3: Figure S6.

Supplementary Material 4: Detailed information about Patient 1 and Patient 2. Supplementary materials and methods.

Supplementary Material 5: Table S1. Analysis of the studdied variants.

## Data Availability

No datasets were generated or analysed during the current study.

## Abbreviations

AM: Antisense molecule
BS: Background splicing
circRNA: Circular RNA
modU7snRNA: Modified U7 small nuclear RNA
MPS IVA: Mucopolysaccharidosis Type IVA
PE: Pseudoexon
