# Supplementary material for: Clinically relevant pseudoexons of the GALNS gene and their antisense-based correction
Source: Mol Med. 2025 May 17;31:196. doi: 10.1186/s10020-025-01243-0 (PMC12085818; doi:10.1186/s10020-025-01243-0)
Supplement: Supplementary file 1 — Supplementary Material 1: Figure S1. [file 10020_2025_1243_MOESM1_ESM.pdf]

Figure S1

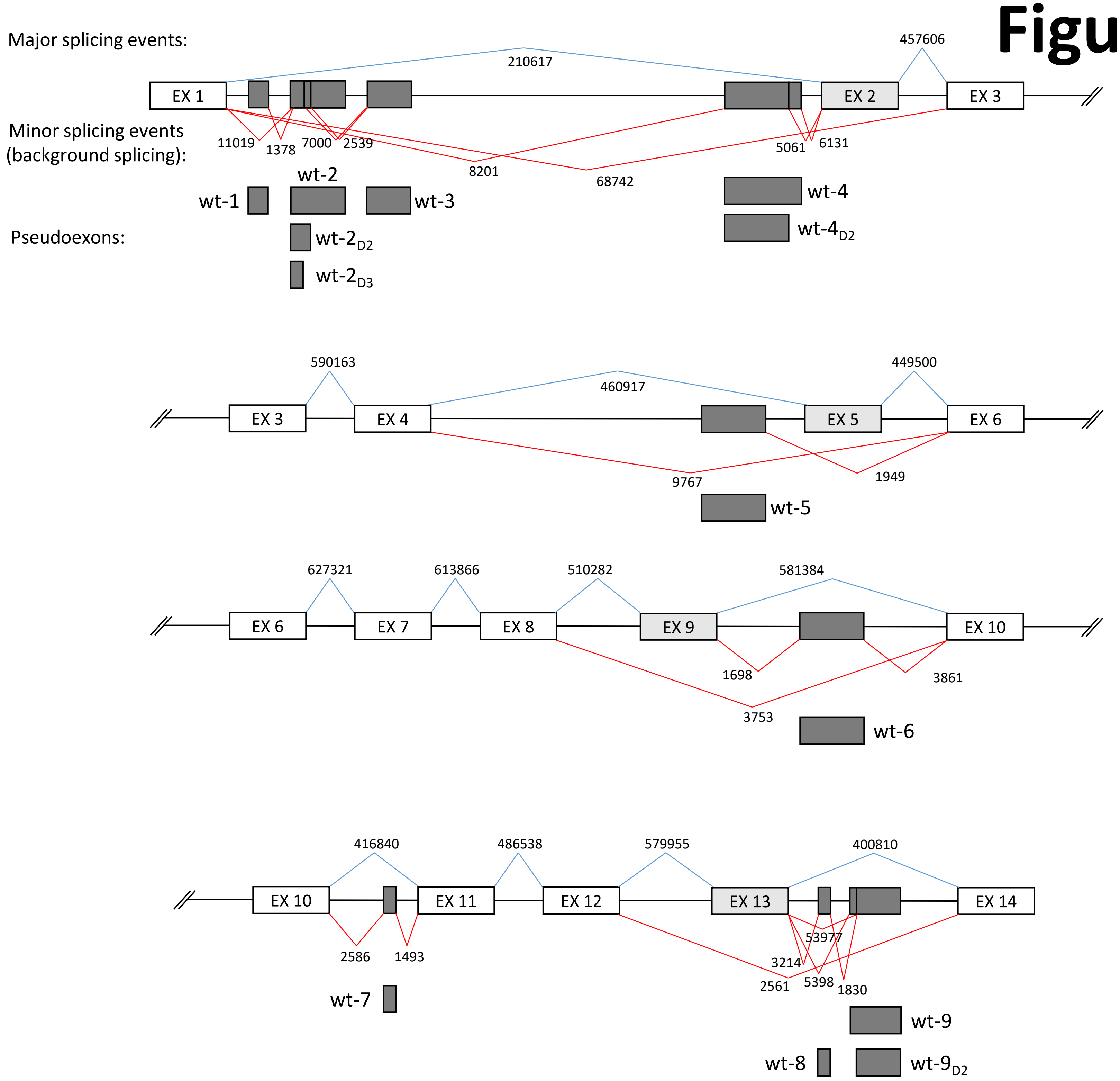

Pseudoexon	Size (bp)	Ensemble transcript	Cordinates (hg38)
wt-1	125	ENST00000562831.1	chr16:88856451-88856575
wt-2	549	ENST00000569433.1	chr16:88855753-88856301
wt-2_{D2}	111		chr16:88856191-88856301
wt-2_{D3}	95	ENST00000568311.1	chr16:88856207-88856301
wt-3	395	ENST00000568311.1	chr16:88855128-88855522
wt-4	223		chr16:88842977-88843199
wt-4_{D2}	170	ENST00000568613.5<br>ENST00000565364.1	chr16:88843030-88843199
wt-5	?		chr16:88838545 – 5’SS
wt-6	376		chr16:88828017-88828392
wt-7	209		chr16:88825471-88825679
wt-8	65		chr16:88817553-88817617
wt-9	824		chr16:88816633-88817456
wt-9_{D2}	785		chr16:88816633-88817417
