# Supplementary material for: Clinically relevant pseudoexons of the GALNS gene and their antisense-based correction
Source: Mol Med. 2025 May 17;31:196. doi: 10.1186/s10020-025-01243-0 (PMC12085818; doi:10.1186/s10020-025-01243-0)
Supplement: Supplementary file 2 — Supplementary Material 2: Figures S2-S5. [file 10020_2025_1243_MOESM2_ESM.pdf]

Figure S2

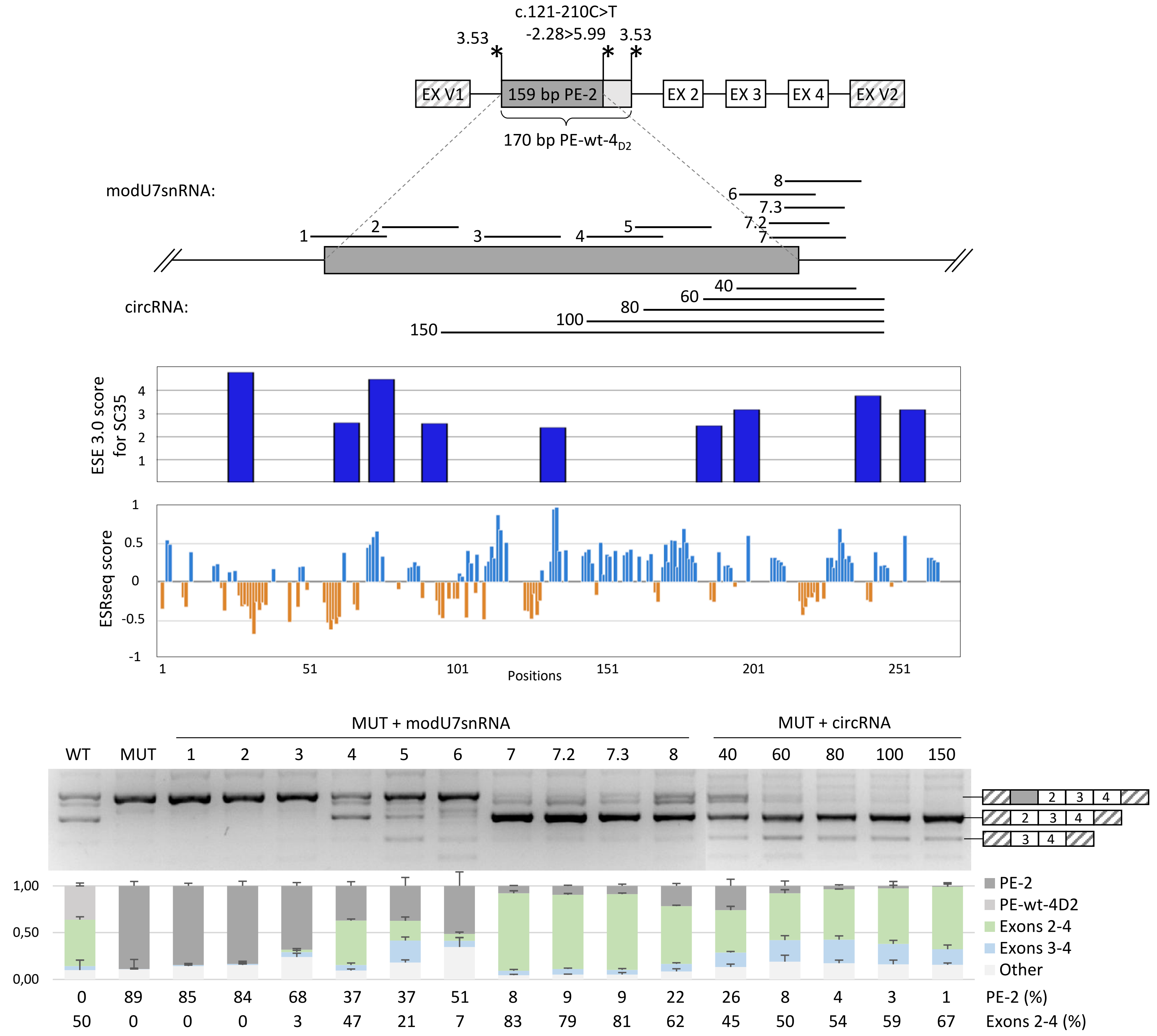

AM	Target	Length (bp)
U7-1	AAATAGATGCACGTGTTTAGAGGCC	25
U7-2	GTTTAGAGGCCAGCTCCTGCGAGCA	25
U7-3	TGGGAGGAGGCAGACGGACACATTC	25
U7-4	GACCAAGCAGAAGTGGAGGCGAAAG	25
U7-5	GCGAAAGACAGCGTGGGCTGACCGT	25
U7-6	CCCATCGTGCACGCACGCAGGTGAT	25
U7-7	ACGCACGCAGGTGATGCTGAGGTTT	25
U7-7.2	ACGCACGCAGGTGATGCTGA	20
U7-7.3	CGCAGGTGATGCTGAGGTTT	20
U7-8	GGTGATGCTGAGGTTTGGGCTGGCG	25
circ-40	CATCGTGCACGCACGCAGGTGATGCTGAGGTTTGGGCTGG	40
circ-60	CGTGGAGCGGCCCCATCGTGCACGCACGCAGGTGATGCTGAGGTTTGGGCTGGCGTGGAG	60
circ-80	GAAAGACAGCGTGGGCTGACCGTGGAGCGGCCCCATCGTGCACGCACGCAGGTGATGCTGAGGTTTGGGCTGGCGTGGAG	80
circ-100	GACCAAGCAGAAGTGGAGGCGAAAGACAGCGTGGGCTGACCGTGGAGCGGCCCCATCGTGCACGCACGCAGGTGATGCTGAGGTTTGGGCTGGC GTGGAG	100
circ-150	ATGCTGGTGTCAGGCTGGGAGGAGGCAGACGGACACATTCCCCACCTGAAGACCAAGCAGAAGTGGAGGCGAAAGACAGCGTGGGCTGACCGTG GAGCGGCCCCATCGTGCACGCACGCAGGTGATGCTGAGGTTTGGGCTGGCGTGGAG	150

Figure S3

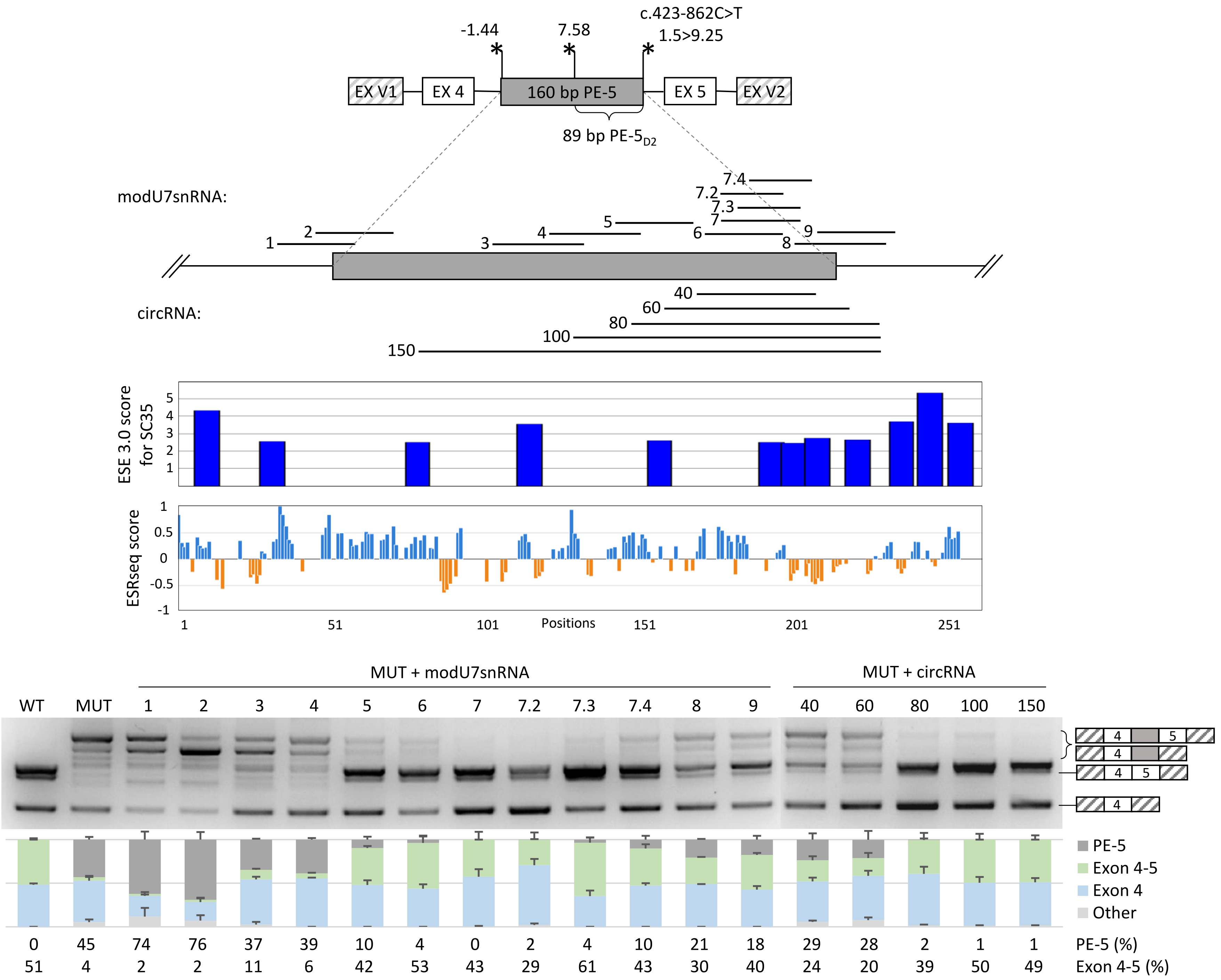

AM	Target	Length (bp)
U7-1	CTGCAGAAGAACTTTCCTCAGACGA	25
U7-2	TCCTCAGACGACACGCAACTGACGT	25
U7-3	TCATACCACCTCTCCTGCAGCCCGTTCTGA	30
U7-4	GCAGCCCGTTCTGAAGAGCAGCCGCAAGCT	30
U7-5	GCAAGCTGGATTCGTTCCAGGACAG	25
U7-6	AGGCTGCGCAAGGAAACCCGGACAG	25
U7-7	GCGCAAGGAAACCCGGACAGCTGGC	25
U7-7.2	GCGCAAGGAAACCCGGACAG	20
U7-7.3	AGGAAACCCGGACAGCTGGC	20
U7-7.4	ACCCGGACAGCTGGCCTGGG	20
U7-8	CTGGGGGCCAGGGTGAGTGGGCACAGAGGT	30
U7-9	CAGGGTGAGTGGGCACAGAGGTGTG	25
circ-40	AGGACAGGCTGCGCAAGGAAACCCGGACAGCTGGCCTGGG	40
circ-60	GTTCCAGGACAGGACAGGCTGCGCAAGGAAACCCGGACAGCTGGCCTGGGGGCCAGGGTG	60
circ-80	AGCTGGATTCGTTCCAGGACAGGACAGGCTGCGCAAGGAAACCCGGACAGCTGGCCTGGGGGCCAGGGTGAGTGGGCACA	80
circ-100	CGTTCTGAAGAGCAGCCGCAAGCTGGATTCGTTCCAGGACAGGACAGGCTGCGCAAGGAAACCCGGACAGCTGGCCTGGGGGCCAGGGTGAGTGGGCACA	100
circ-150	CCGCCCACGGCCGTGCCTAGAAGCCATCTCATACCACCTCTCCTGCAGCCCGTTCTGAAGAGCAGCCGCAAGCTGGATTCGTTCCAGGACAGGACAGGCTG CGCAAGGAAACCCGGACAGCTGGCCTGGGGGCCAGGGTGAGTGGGCACA	150

Figure S4

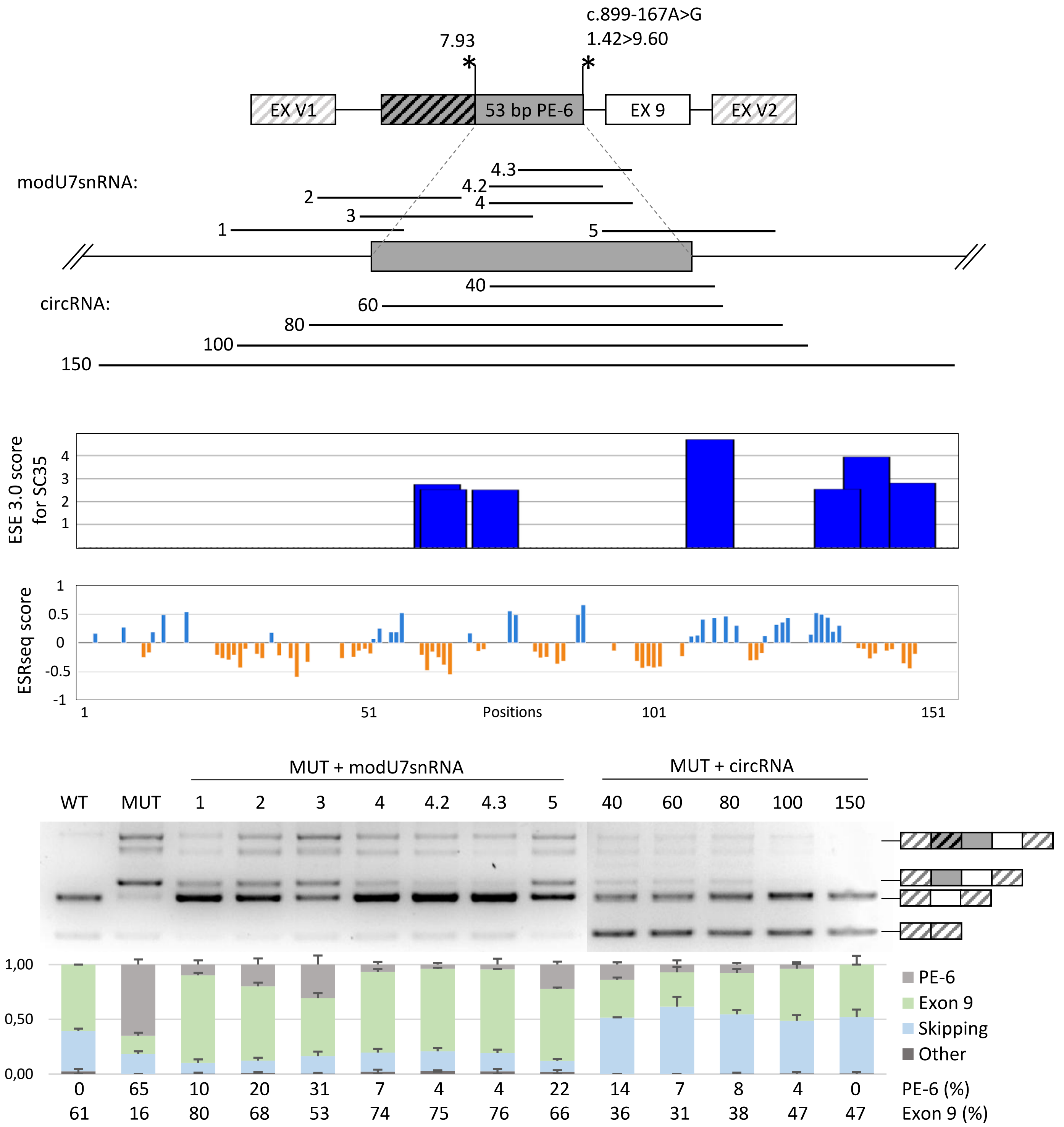

U7	Target	Length (bp)
U7-1	TATGGTTGTGTTTCCATTTCCTCAGTGGGC	30
U7-2	ATTTCCTCAGTGGGCATGATGTCCC	25
U7-3	CAGTGGGCATGATGTCCCCACTTTCTCCAG	30
U7-4	TTCTCCAGGAGACTTTGCAGCCGCG	25
U7-4.2	TTCTCCAGGAGACTTTGCAG	20
U7-4.3	CAGGAGACTTTGCAGCCGCG	20
circ-5	CCGCGGCATCTCAGGTGAGCCCCTGGAGAG	30
circ-40	TTCTCCAGGAGACTTTGCAGCCGCGGCATCTCAGGTGAGC	40
circ-60	TGGGCATGATGTCCCCACTTTCTCCAGGAGACTTTGCAGCCGCGGCATCTCAGGTGAGCC	60
circ-80	ATTTCCTCAGTGGGCATGATGTCCCCACTTTCTCCAGGAGACTTTGCAGCCGCGGCATCTCAGGTGAGCCCCTGGAGAGC	80
circ-100	TATGGTTGTGTTTCCATTTCCTCAGTGGGCATGATGTCCCCACTTTCTCCAGGAGACTTTGCAGCCGCGGCATCTCAGGTGAGCCCCTGG AGAGCCACCC	100
circ-150	CCTCTCTGAGTCTTGCTGACACCGTATGGTTGTGTTTCCATTTCCTCAGTGGGCATGATGTCCCCACTTTCTCCAGGAGACTTTGCAGCC GCGGCATCTCAGGTGAGCCCCTGGAGAGCCACCCCGAGGCTCGGATCATGCACTCCAGCC	150

Figure S5

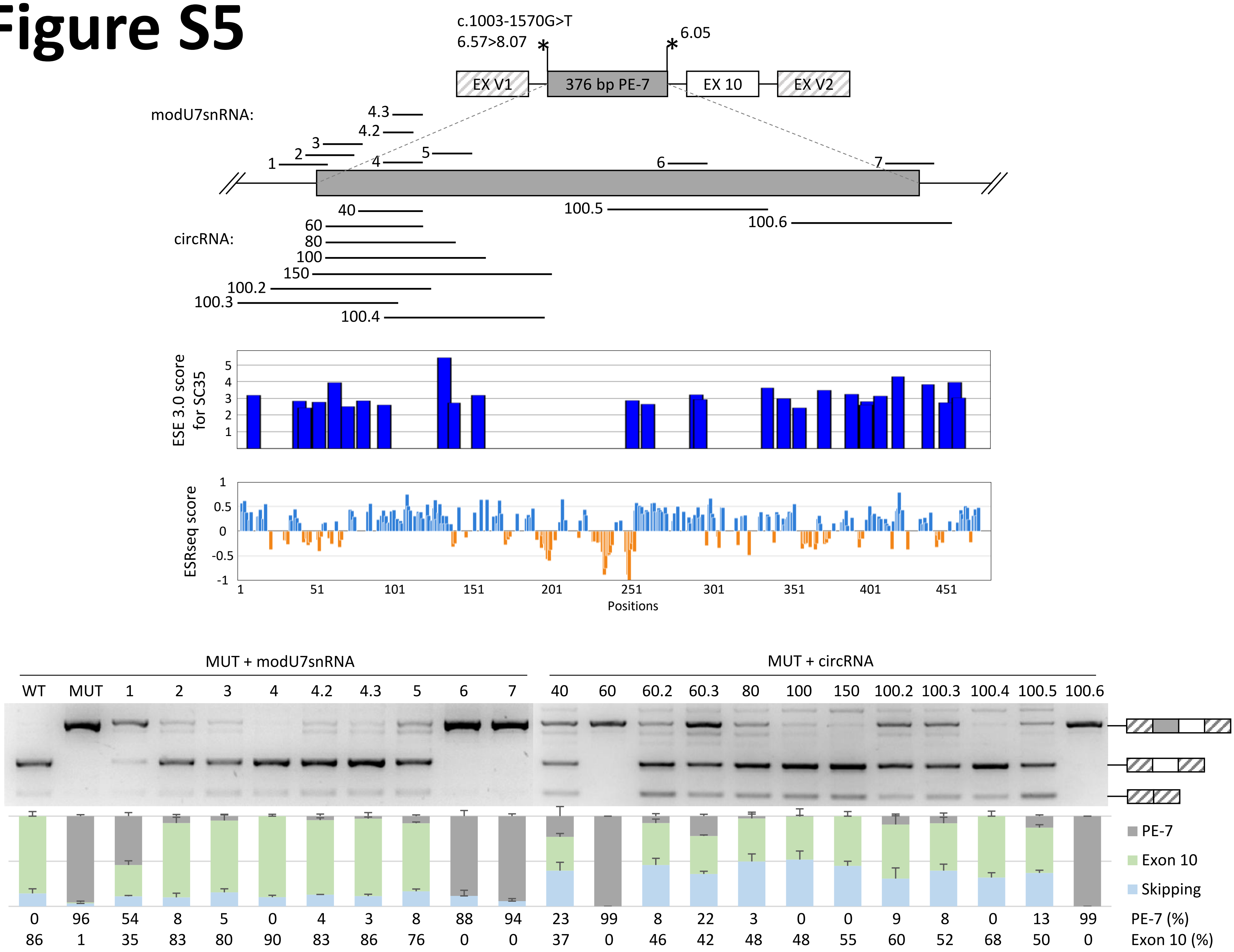

U7	Target	bp
U7-1	acggtttcattgacttctcccacagCCGCA	30
U7-2	cagCCGCAGCTGCCCACTGCATTTCTGGAT	30
U7-3	GCCCACTGCATTTCTGGATGTTTCT	25
U7-4	TCGCTGCCTGGAGAGTTCTTCGAAA	25
U7-4.2	TCGCTGCCTGGAGAGTTCTT	20
U7-4.3	GCCTGGAGAGTTCTTCGAAA	20
U7-5	CACGAGGACCCTGCCTGTGCTGACA	25
U7-6	GGAAGCAGTCGCACAGACACTGCTG	25
U7-7	GCGGCTGCAGCGCGGAgtgagtgtccctgt	30
circ-40	GCCCACTGCATTTCTGGATGTTTCTGGAAGGCATCGCTGC	40
circ-60	GCCCACTGCATTTCTGGATGTTTCTGGAAGGCATCGCTGCCTGGAGAGTTCTTCGAAAAC	60
circ-60.2	CAGCTGCCCACTGCATTTCTGGATGTTTCTGGAAGGCATCGCTGCCTGGAGAGTTCTTCG	60
circ-60.3	CTGCATTTCTGGATGTTTCTGGAAGGCATCGCTGCCTGGAGAGTTCTTCGAAAACGCTCC	60
circ-80	GCCCACTGCATTTCTGGATGTTTCTGGAAGGCATCGCTGCCTGGAGAGTTCTTCGAAAACGCTCCACGAGGACCCTGCCT	80
circ-100	GCCCACTGCATTTCTGGATGTTTCTGGAAGGCATCGCTGCCTGGAGAGTTCTTCGAAAACGCTCCACGAGGACCCTGCCTGTGCTGACACCACTCCCGCC	100
circ-150	cagCCGCAGCTGCCCACTGCATTTCTGGATGTTTCTGGAAGGCATCGCTGCCTGGAGAGTTCTTCGAAAACGCTCCACGAGGACCCTGCCTGTGCTGACACCACT CCCGCCCGCCCGCCCTGCCGGCACCACAAGCACTGCTCCGCAGTT	150
circ-100.2	attgacttctcccacagCCGCAGCTGCCCACTGCATTTCTGGATGTTTCTGGAAGGCATCGCTGCCTGGAGAGTTCTTCGAAAACGCTCCACGAGGACCC	100
circ-100.3	tctcgaaagcgctgataacggtttcattgacttctcccacagCCGCAGCTGCCCACTGCATTTCTGGATGTTTCTGGAAGGCATCGCTGCCTGGAGAGTT	100
circ-100.4	AGTTCTTCGAAAACGCTCCACGAGGACCCTGCCTGTGCTGACACCACTCCCGCCCGCCCGCCCTGCCGGCACCACAAGCACTGCTCCGCAGTTATTTTTT	100
circ-100.5	ATTCTGTCCTTTAAAGGACACGGGAAGGACGGGAAGCAGTCGCACAGACACTGCTGACCAGACCTGGGGAAGCTTCTCACCTTCTGCAAAGCTTCCTGAA	100
circ-100.6	CGCTGAGCTTTGCATTCCCTGCCCCACACTGGGCGCTGCAGACCCCACATCCCGTGAGCGGCTGCAGCGCGGAgtgagtgtccctgtgaggccagggctc	100
