# Supplementary figures and images for: Clinically relevant pseudoexons of the GALNS gene and their antisense-based correction
Source: Mol Med. 2025 May 17;31:196. doi: 10.1186/s10020-025-01243-0 (PMC12085818; doi:10.1186/s10020-025-01243-0)

# Figure S6

Uncropped gel images from figure 1f.

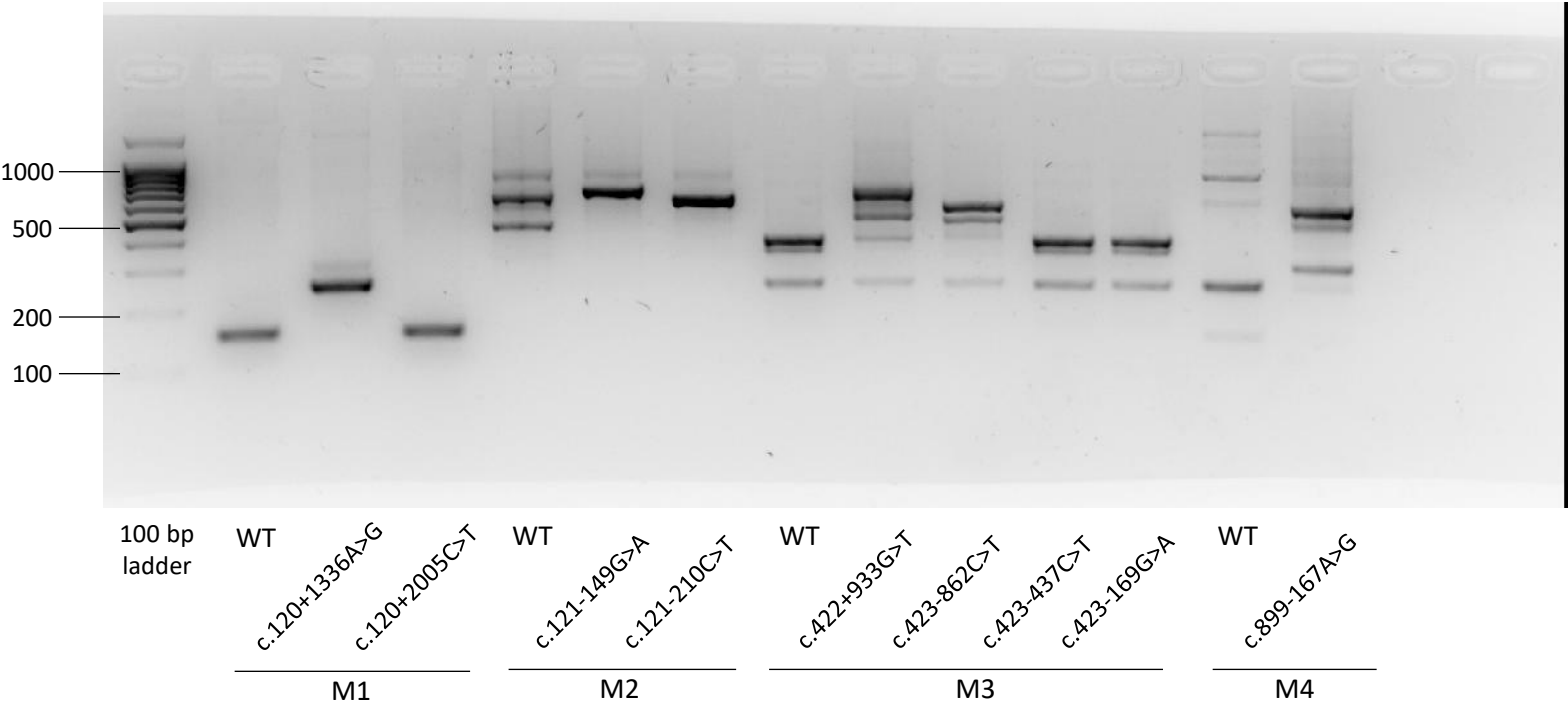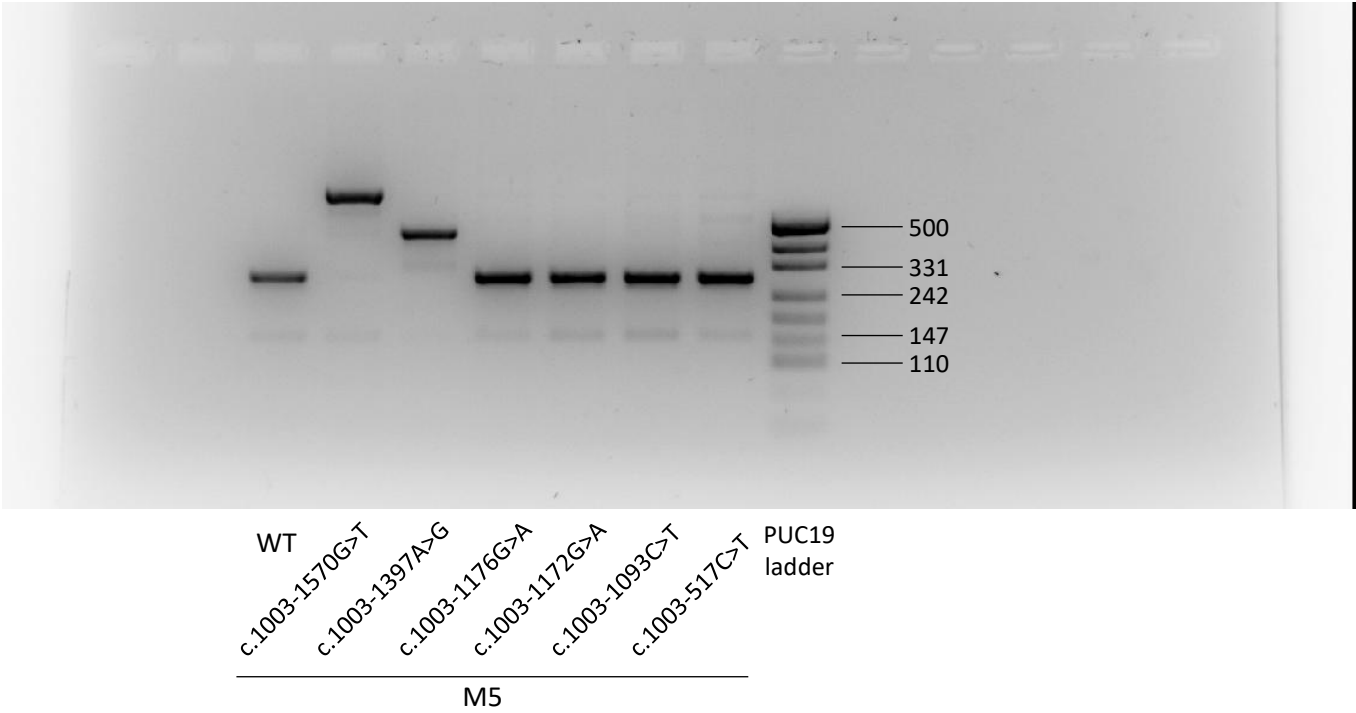

Supplement: Supplementary file 3 — Supplementary Material 3: Figure S6. [file 10020_2025_1243_MOESM3_ESM.pdf]
