# Supplementary material for: Clinically relevant pseudoexons of the GALNS gene and their antisense-based correction
Source: Mol Med. 2025 May 17;31:196. doi: 10.1186/s10020-025-01243-0 (PMC12085818; doi:10.1186/s10020-025-01243-0)
Supplement: Supplementary file 4 — Supplementary Material 4: Detailed information about Patient 1 and Patient 2. Supplementary materials and methods. [file 10020_2025_1243_MOESM4_ESM.pdf]

Supplementary information 1.

Detailed information about Patient 1 and Patient 2.

Patient 1 is an 8-year-old boy. The diagnosis of MPS IVA was established at the age of 4 based on the typical clinical, radiological, and biochemical findings (a severe decrease in the activity of the enzyme N-acetylgalactosamine-6-sulfatase in dried blood spots).

At birth, the weight was 3950 g, the length was 58 cm, with an early development appropriate for patient's age. Patient started walking at 11 months. By the age of 1 year, he developed valgus feet deformity and prominent, wide lower ribs. At 2.5 years, he had a pectus carinatum (pigeon chest), and by 4 years, he developed X-shaped deformity of the lower limbs. His growth rate slowed down since 2.5 years old. The diagnosis of spondyloepiphyseal dysplasia was established at 4.5 years old. At the age of 3, he underwent surgical correction for the deformity of the lower limbs.

At the time of examination at 8 years, the height was 111 cm, the weight was 22 kg. Main clinical features include: short stature. kyphoscoliosis, short neck, truncal shortening, relatively long limbs, pectus carinatum, wide lower thoracic aperture, and thickened lower ribs, contractures of major joints, except for hypermobility of the wrists and fingers, ulnar deviation of the wrists, gait with valgus positioning of the knees and ankles.

NGS sequencing of the *GALNS* gene (gene panel sequencing) failed to detect any pathogenic variants, so the patient's fresh blood sample was collected and subjected to RNA analysis. Results of the *GALNS* mRNA analysis demonstrated an inclusion of PE-5 (Figure 1b), activated by the c.423-862C>T variant. The variant was identified in homozygous state in patient and in heterozygous state in patients' parents.

Patient 2 is a 8-year-old boy from consanguineous marriage. The child is from the second pregnancy, which was complicated by a threat of miscarriage in the third trimester.

The birth weight was 3500 g, and the length was 54 cm. Patient held his head up from 2 months, sat at 10 months, and began walking at 14 months, psychomotor development was age-appropriate. From the mother's words, from the age of 1.5 years, deformities of the chest and lower limbs were noted. From age 6, he was under observation in the traumatology and orthopedic department with a diagnosis of valgus deformity of the knee joints and pectus carinatum. In the

same year, he underwent varus osteotomy of the lower third of both femurs with placement of metal structures, which were subsequently removed.

At the time of examination at age 8, the following clinical features were observed: multiple dysostosis syndrome, basilar foramen stenosis, pectus carinatum, prominent lower thoracic aperture, gargoyle-like facial features, hypertelorism, epicanthus, smooth philtrum, broad nasal bridge, valgus deformity of the lower extremities. There was pronounced congenital hypoplasia of the odontoid process with marked instability of the bony elements of the craniovertebral junction. Severe narrowing of the foramen magnum was noted, with compression of the medulla oblongata at this level. Vertebral body deformities resembled "fish vertebrae". Mild aortic valve insufficiency (grade I), aortic valve stenosis, aortic root dilatation, and grade I corneal clouding were also present. Patient also has a 5-year-old brother with the similar clinical phenotype.

At the age 7, the family was referred to our center due to suspected hereditary disease. For both patients, the dried blood spots were initially delivered to our Centre, so we performed measurement of the *GALNS* enzyme activity by tandem mass spectrometry. The enzyme activity was severely reduced, so the DNA was extracted from dried blood spots and subjected to Sanger sequencing of the *GALNS* gene. After we fail to identify biallelic variants in the *GALNS* gene, the fresh blood sample of patient 2 was delivered to our Centre for RNA analysis. The results of the RNA analysis demonstrated an insertion of PE-7 activated by the c.1003-1570G>T variant. The variant was identified in homozygous state in patient and his brother, and in heterozygous state in patients' parents.

Supplementary materials and methods

Measurement of the *GALNS* enzyme activity was performed by tandem mass spectrometry, as described previously [1].

1. Bychkov, I., et al., *Functional Analysis of Complex Structural and Splice-Altering Variants in the ARSB Gene Towards the Personalized Antisense-Based Therapy for Mucopolysaccharidosis Type VI Patients.* Human Mutation, 2025. **2025**(1): p. 2250030.
